# Bis(di-2-pyridylmethane­diol-κ^3^ *N*,*O*,*N*′)copper(II) bis­(tetra­fluorido­borate) dihydrate

**DOI:** 10.1107/S1600536809016973

**Published:** 2009-05-14

**Authors:** Krystal L. Brown, Guy Crundwell, Barry L. Westcott

**Affiliations:** a Department of Chemistry and Biochemistry, Central Connecticut State University, New Britain, CT 06050, USA

## Abstract

The title complex, [Cu(C_11_H_10_N_2_O_2_)_2_](BF_4_)_2_·2H_2_O, was isolated as a dihydrate from a 1:2 molar mixture of copper(II) tetra­fluoridoborate hexa­hydrate with di-2-pyridyl ketone in aqueous solution. The centrosymmetric complex cation is structurally similar to that found in previously reported salts and exhibits Cu—O bonds deviating by 25 degrees from an octa­hedral geometry by the so-called ‘off-axis angle’ distortion. The BF_4_^−^ anion exhibits a two site disorder of the fluorine atoms [ratio 0.210 (8):0.790 (8)].

## Related literature

For related structures, see: Wang *et al.* (1986 ▶); Tangoulis *et al.* (1997 ▶); Yang *et al.* (1998 ▶); Tong *et al.* (1998 ▶); Serna *et al.* (1999 ▶); Reinoso *et al.* (2003 ▶); Li *et al.* (2005 ▶).
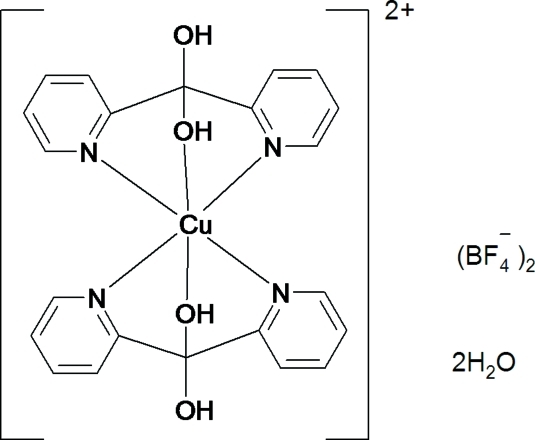


## Experimental

### 

#### Crystal data

[Cu(C_11_H_10_N_2_O_2_)_2_](BF_4_)_2_·2H_2_O
*M* *_r_* = 677.61
Monoclinic, 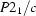

*a* = 7.8147 (2) Å
*b* = 14.4225 (4) Å
*c* = 12.1840 (3) Å
β = 101.160 (3)°
*V* = 1347.26 (6) Å^3^
*Z* = 2
Mo *K*α radiation
μ = 0.91 mm^−1^
*T* = 293 K
0.8 × 0.6 × 0.6 mm

#### Data collection

Oxford Diffraction Sapphire CCD diffractometer
Absorption correction: multi-scan *SCALE3 ABSPACK* in *CrysAlis RED* (Oxford Diffraction, 2006 ▶) *T*_min_ = 0.474, *T*_max_ = 0.579
25175 measured reflections
5349 independent reflections
4145 reflections with *I* > 2σ(*I*)
*R*_int_ = 0.036

#### Refinement

*R*[*F* ^2^ > 2σ(*F* ^2^)] = 0.055
*wR*(*F* ^2^) = 0.192
*S* = 1.21
5349 reflections
219 parameters
10 restraints
H atoms treated by a mixture of independent and constrained refinement
Δρ_max_ = 0.72 e Å^−3^
Δρ_min_ = −1.03 e Å^−3^

### 

Data collection: *CrysAlis CCD* (Oxford Diffraction, 2006 ▶); cell refinement: *CrysAlis RED* (Oxford Diffraction, 2006 ▶); data reduction: *CrysAlis RED*; program(s) used to solve structure: *SHELXS97* (Sheldrick, 2008 ▶); program(s) used to refine structure: *SHELXL97* (Sheldrick, 2008 ▶); molecular graphics: *ORTEP-3* (Farrugia, 1997 ▶); software used to prepare material for publication: *SHELXTL* (Sheldrick, 2008 ▶).

## Supplementary Material

Crystal structure: contains datablocks I, global. DOI: 10.1107/S1600536809016973/fj2210sup1.cif

Structure factors: contains datablocks I. DOI: 10.1107/S1600536809016973/fj2210Isup2.hkl

Additional supplementary materials:  crystallographic information; 3D view; checkCIF report

## Figures and Tables

**Table 1:** Selected geometric parameters (Å, °)

Cu1—N1^i^	2.0099 (19)
Cu1—N1	2.0099 (19)
Cu1—N2	2.0146 (19)
Cu1—N2^i^	2.0147 (19)
Cu1—O1	2.4312 (17)
Cu1—O1^i^	2.4312 (17)

Symmetry code: (i) 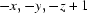.

**Table 2:** Hydrogen-bond geometry (Å, °)

*D*—H⋯*A*	*D*—H	H⋯*A*	*D*⋯*A*	*D*—H⋯*A*
O2—H2⋯O3	0.82	1.87	2.686 (3)	172

Symmetry codes: .

**Fig. 1.**
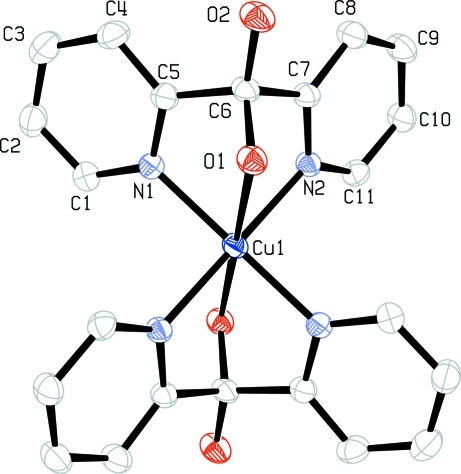
ORTEP representation of the title complex. Thermal ellipsoids are drawn at the 30% probability level and H atoms have been omitted for clarity.

## supplementary crystallographic information

### Experimental

All chemicals and reagents were purchased from Aldrich and used as received. di-2-pyridyl ketone (2 mmol) and copper(II) tetrafluorborate hexahydrate (1 mmol) were combined in 40 ml of water and stirred for 30 minutes. The resulting violet crystals were isolated after 48 h by slow evaporation of the solution.

### Refinement

For structure solution, direct methods were used to locate the initial structural model that consisted of all non-hydrogen atoms.  All ligand-based H atoms were added during the refinement stage at idealized positions. Water-based H atoms were found during subsequent cycles from difference maps and their bond lengths to oxygen were free to refine. All H atoms were refined isotropically and all non-hydrogen atoms were refined anisotropically.

### Crystal data

**Table:**

[Cu(C_11_H_10_N_2_O_2_)_2_](BF_4_)_2_·2H_2_O	*F*(000) = 686
*M**_r_* = 677.61	*D*_x_ = 1.670 Mg m^−^^3^
Monoclinic, *P*2_1_/*c*	Mo *K*α radiation, λ = 0.71073 Å
Hall symbol: -P 2ybc	Cell parameters from 5349 reflections
*a* = 7.8147 (2) Å	θ = 3.8–32.0°
*b* = 14.4225 (4) Å	µ = 0.91 mm^−^^1^
*c* = 12.1840 (3) Å	*T* = 293 K
β = 101.160 (3)°	Parallelpiped, violet
*V* = 1347.26 (6) Å^3^	0.8 × 0.6 × 0.6 mm
*Z* = 2	

### Data collection

**Table:**

Oxford Diffraction Sapphire CCD diffractometer	5349 independent reflections
Radiation source: fine-focus sealed tube	4145 reflections with *I* > 2σ(*I*)
graphite	*R*_int_ = 0.036
ω scans	θ_max_ = 34.7°, θ_min_ = 3.9°
Absorption correction: multi-scan SCALE3 ABSPACK in *CrysAlis RED* (Oxford Diffraction, 2006)	*h* = −12→11
*T*_min_ = 0.474, *T*_max_ = 0.579	*k* = −22→22
25175 measured reflections	*l* = −18→19

### Refinement

**Table:**

Refinement on *F*^2^	Primary atom site location: structure-invariant direct methods
Least-squares matrix: full	Secondary atom site location: difference Fourier map
*R*[*F*^2^ > 2σ(*F*^2^)] = 0.055	Hydrogen site location: inferred from neighbouring sites
*wR*(*F*^2^) = 0.192	H atoms treated by a mixture of independent and constrained refinement
*S* = 1.21	*w* = 1/[σ^2^(*F*_o_^2^) + (0.0959*P*)^2^ + 1.*P*] where *P* = (*F*_o_^2^ + 2*F*_c_^2^)/3
5349 reflections	(Δ/σ)_max_ < 0.001
219 parameters	Δρ_max_ = 0.72 e Å^−^^3^
10 restraints	Δρ_min_ = −1.03 e Å^−^^3^

### Special details

**Table:**

Geometry. All e.s.d.'s (except the e.s.d. in the dihedral angle between two l.s. planes) are estimated using the full covariance matrix. The cell e.s.d.'s are taken into account individually in the estimation of e.s.d.'s in distances, angles and torsion angles; correlations between e.s.d.'s in cell parameters are only used when they are defined by crystal symmetry. An approximate (isotropic) treatment of cell e.s.d.'s is used for estimating e.s.d.'s involving l.s. planes.
Refinement. Hydrogen atoms were included in calculated positions for the ring carbons on the dpk ligand (0.93Å for *sp^2^* carbons) and were included in the refinement in riding motion approximation with U_iso_ = 1.2U_eq_ of the carrier atom for *sp^2^* carbons. Oxygen hydrogens were found in difference maps.Refinement of *F*^2^ against ALL reflections. The weighted *R*-factor *wR* and goodness of fit *S* are based on *F*^2^, conventional *R*-factors *R* are based on *F*, with *F* set to zero for negative *F*^2^. The threshold expression of *F*^2^ > σ(*F*^2^) is used only for calculating *R*-factors(gt) *etc*. and is not relevant to the choice of reflections for refinement. *R*-factors based on *F*^2^ are statistically about twice as large as those based on *F*, and *R*- factors based on ALL data will be even larger.

### Fractional atomic coordinates and isotropic or equivalent isotropic displacement parameters (Å^2^)

**Table:**

	*x*	*y*	*z*	*U*_iso_*/*U*_eq_	Occ. (<1)
Cu1	0.0000	0.0000	0.5000	0.02185 (12)	
C1	0.2975 (3)	0.04790 (18)	0.3891 (2)	0.0289 (5)	
H1	0.2514	0.0035	0.3363	0.035*	
C2	0.4472 (4)	0.0936 (2)	0.3777 (2)	0.0335 (5)	
H2A	0.5001	0.0810	0.3172	0.040*	
C3	0.5190 (4)	0.1586 (2)	0.4571 (2)	0.0348 (5)	
H3	0.6217	0.1894	0.4517	0.042*	
C4	0.4334 (3)	0.17694 (18)	0.5455 (2)	0.0310 (5)	
H4	0.4780	0.2203	0.6001	0.037*	
C5	0.2824 (3)	0.12975 (15)	0.55022 (18)	0.0243 (4)	
C6	0.1798 (3)	0.14291 (15)	0.64412 (18)	0.0240 (4)	
C7	0.1983 (3)	0.05478 (15)	0.71483 (18)	0.0228 (4)	
C8	0.2869 (4)	0.05111 (17)	0.8236 (2)	0.0294 (5)	
H8	0.3411	0.1036	0.8587	0.035*	
C9	0.2936 (4)	−0.03360 (19)	0.8802 (2)	0.0322 (5)	
H9	0.3511	−0.0383	0.9543	0.039*	
C10	0.2138 (4)	−0.11005 (17)	0.8244 (2)	0.0289 (4)	
H10	0.2176	−0.1671	0.8604	0.035*	
C11	0.1283 (3)	−0.10113 (16)	0.71447 (19)	0.0250 (4)	
H11	0.0746	−0.1529	0.6772	0.030*	
N1	0.2158 (3)	0.06543 (13)	0.47416 (15)	0.0230 (3)	
N2	0.1204 (3)	−0.02000 (13)	0.65999 (15)	0.0214 (3)	
O1	0.0028 (2)	0.15157 (12)	0.58765 (14)	0.0252 (3)	
O2	0.2355 (3)	0.21705 (12)	0.71285 (15)	0.0310 (4)	
H2	0.2214	0.2651	0.6763	0.047*	
H50	−0.064 (5)	0.153 (3)	0.633 (3)	0.034 (9)*	
H51	0.181 (7)	0.416 (4)	0.599 (4)	0.058 (13)*	
H52	0.133 (6)	0.354 (3)	0.529 (4)	0.044 (11)*	
O3	0.2110 (4)	0.36785 (16)	0.5814 (2)	0.0419 (5)	
B1	0.8127 (4)	0.13674 (19)	0.8480 (2)	0.0302 (5)	0.790 (8)
F1	0.7868 (2)	0.16783 (13)	0.73741 (13)	0.0381 (4)	0.790 (8)
F2	0.8638 (9)	0.0451 (3)	0.8536 (8)	0.0471 (13)	0.790 (8)
F3	0.9557 (7)	0.1892 (2)	0.9056 (3)	0.0674 (14)	0.790 (8)
F4	0.6729 (5)	0.1531 (3)	0.8951 (3)	0.0661 (12)	0.790 (8)
B1B	0.8127 (4)	0.13674 (19)	0.8480 (2)	0.0302 (5)	0.210 (8)
F1B	0.7868 (2)	0.16783 (13)	0.73741 (13)	0.0381 (4)	0.210 (8)
F2B	0.894 (4)	0.0519 (14)	0.854 (3)	0.0471 (13)	0.210 (8)
F3B	0.866 (3)	0.1927 (9)	0.9307 (11)	0.0674 (14)	0.210 (8)
F4B	0.6394 (16)	0.1147 (11)	0.8558 (12)	0.0661 (12)	0.210 (8)

### Atomic displacement parameters (Å^2^)

**Table:**

	*U*^11^	*U*^22^	*U*^33^	*U*^12^	*U*^13^	*U*^23^
Cu1	0.0261 (2)	0.02088 (19)	0.01787 (18)	−0.00313 (13)	0.00248 (13)	−0.00088 (12)
C1	0.0302 (11)	0.0331 (12)	0.0238 (10)	−0.0027 (9)	0.0066 (8)	0.0000 (8)
C2	0.0302 (12)	0.0398 (14)	0.0323 (12)	−0.0002 (10)	0.0109 (9)	0.0049 (10)
C3	0.0271 (12)	0.0358 (13)	0.0423 (14)	−0.0049 (9)	0.0088 (10)	0.0050 (11)
C4	0.0308 (12)	0.0257 (10)	0.0350 (12)	−0.0068 (9)	0.0024 (9)	0.0008 (9)
C5	0.0271 (10)	0.0209 (9)	0.0234 (9)	−0.0013 (7)	0.0016 (7)	0.0019 (7)
C6	0.0313 (11)	0.0184 (8)	0.0210 (9)	−0.0036 (7)	0.0016 (7)	−0.0010 (7)
C7	0.0248 (10)	0.0206 (9)	0.0223 (9)	−0.0026 (7)	0.0030 (7)	−0.0012 (7)
C8	0.0352 (12)	0.0263 (10)	0.0246 (10)	−0.0040 (9)	0.0006 (8)	−0.0005 (8)
C9	0.0397 (13)	0.0323 (12)	0.0222 (10)	−0.0016 (10)	0.0004 (9)	0.0025 (9)
C10	0.0351 (12)	0.0249 (10)	0.0259 (10)	−0.0006 (9)	0.0037 (9)	0.0046 (8)
C11	0.0259 (10)	0.0206 (9)	0.0285 (10)	−0.0024 (7)	0.0053 (8)	0.0008 (7)
N1	0.0239 (8)	0.0236 (8)	0.0213 (8)	−0.0030 (6)	0.0036 (6)	0.0005 (6)
N2	0.0241 (8)	0.0198 (7)	0.0201 (7)	−0.0016 (6)	0.0035 (6)	−0.0003 (6)
O1	0.0270 (8)	0.0252 (7)	0.0227 (7)	0.0009 (6)	0.0026 (6)	0.0011 (6)
O2	0.0455 (11)	0.0198 (7)	0.0257 (8)	−0.0050 (7)	0.0019 (7)	−0.0033 (6)
O3	0.0584 (14)	0.0259 (9)	0.0390 (11)	0.0041 (9)	0.0035 (10)	0.0029 (8)
B1	0.0412 (15)	0.0245 (11)	0.0250 (11)	0.0042 (10)	0.0064 (10)	0.0002 (9)
F1	0.0457 (10)	0.0417 (9)	0.0268 (7)	0.0064 (7)	0.0072 (6)	0.0068 (6)
F2	0.069 (3)	0.0238 (11)	0.0477 (10)	0.0088 (16)	0.010 (2)	0.0005 (10)
F3	0.094 (3)	0.0412 (13)	0.0503 (17)	−0.0201 (19)	−0.0284 (19)	0.0030 (12)
F4	0.084 (2)	0.072 (3)	0.054 (2)	0.0369 (19)	0.0436 (18)	0.0173 (17)
B1B	0.0412 (15)	0.0245 (11)	0.0250 (11)	0.0042 (10)	0.0064 (10)	0.0002 (9)
F1B	0.0457 (10)	0.0417 (9)	0.0268 (7)	0.0064 (7)	0.0072 (6)	0.0068 (6)
F2B	0.069 (3)	0.0238 (11)	0.0477 (10)	0.0088 (16)	0.010 (2)	0.0005 (10)
F3B	0.094 (3)	0.0412 (13)	0.0503 (17)	−0.0201 (19)	−0.0284 (19)	0.0030 (12)
F4B	0.084 (2)	0.072 (3)	0.054 (2)	0.0369 (19)	0.0436 (18)	0.0173 (17)

### Geometric parameters (Å, °)

**Table:**

Cu1—N1^i^	2.0099 (19)	C6—C7	1.527 (3)
Cu1—N1	2.0099 (19)	C7—N2	1.350 (3)
Cu1—N2	2.0146 (19)	C7—C8	1.372 (3)
Cu1—N2^i^	2.0147 (19)	C8—C9	1.399 (4)
Cu1—O1	2.4312 (17)	C8—H8	0.9300
Cu1—O1^i^	2.4312 (17)	C9—C10	1.379 (4)
C1—N1	1.343 (3)	C9—H9	0.9300
C1—C2	1.373 (4)	C10—C11	1.382 (3)
C1—H1	0.9300	C10—H10	0.9300
C2—C3	1.385 (4)	C11—N2	1.341 (3)
C2—H2A	0.9300	C11—H11	0.9300
C3—C4	1.399 (4)	O1—H50	0.83 (4)
C3—H3	0.9300	O2—H2	0.8200
C4—C5	1.373 (3)	O3—H51	0.78 (5)
C4—H4	0.9300	O3—H52	0.82 (5)
C5—N1	1.343 (3)	B1—F4	1.350 (4)
C5—C6	1.531 (3)	B1—F2	1.378 (5)
C6—O2	1.376 (3)	B1—F1	1.397 (3)
C6—O1	1.427 (3)	B1—F3	1.417 (5)
			
N1^i^—Cu1—N1	179.999 (1)	O1—C6—C5	104.48 (17)
N1^i^—Cu1—N2	91.74 (8)	C7—C6—C5	108.21 (18)
N1—Cu1—N2	88.27 (8)	N2—C7—C8	122.8 (2)
N1^i^—Cu1—N2^i^	88.26 (8)	N2—C7—C6	113.67 (19)
N1—Cu1—N2^i^	91.73 (8)	C8—C7—C6	123.5 (2)
N2—Cu1—N2^i^	180.0	C7—C8—C9	118.3 (2)
N1^i^—Cu1—O1	106.84 (7)	C7—C8—H8	120.9
N1—Cu1—O1	73.16 (7)	C9—C8—H8	120.9
N2—Cu1—O1	74.99 (7)	C10—C9—C8	119.0 (2)
N2^i^—Cu1—O1	105.01 (7)	C10—C9—H9	120.5
N1^i^—Cu1—O1^i^	73.16 (7)	C8—C9—H9	120.5
N1—Cu1—O1^i^	106.84 (7)	C11—C10—C9	119.4 (2)
N2—Cu1—O1^i^	105.01 (7)	C11—C10—H10	120.3
N2^i^—Cu1—O1^i^	74.99 (7)	C9—C10—H10	120.3
O1—Cu1—O1^i^	180.00 (8)	N2—C11—C10	121.9 (2)
N1—C1—C2	122.0 (2)	N2—C11—H11	119.1
N1—C1—H1	119.0	C10—C11—H11	119.1
C2—C1—H1	119.0	C5—N1—C1	118.9 (2)
C1—C2—C3	119.5 (2)	C5—N1—Cu1	116.18 (15)
C1—C2—H2A	120.2	C1—N1—Cu1	124.86 (16)
C3—C2—H2A	120.2	C11—N2—C7	118.63 (19)
C2—C3—C4	118.4 (2)	C11—N2—Cu1	124.89 (15)
C2—C3—H3	120.8	C7—N2—Cu1	116.48 (15)
C4—C3—H3	120.8	C6—O1—Cu1	93.35 (12)
C5—C4—C3	118.8 (2)	C6—O1—H50	111 (3)
C5—C4—H4	120.6	Cu1—O1—H50	111 (3)
C3—C4—H4	120.6	C6—O2—H2	109.5
N1—C5—C4	122.3 (2)	H51—O3—H52	103 (5)
N1—C5—C6	114.34 (19)	F4—B1—F2	113.4 (4)
C4—C5—C6	123.3 (2)	F4—B1—F1	112.1 (3)
O2—C6—O1	112.86 (19)	F2—B1—F1	110.0 (4)
O2—C6—C7	108.62 (18)	F4—B1—F3	108.8 (3)
O1—C6—C7	108.78 (18)	F2—B1—F3	107.1 (4)
O2—C6—C5	113.67 (19)	F1—B1—F3	105.0 (3)

Symmetry codes: (i) −*x*, −*y*, −*z*+1.

### Hydrogen-bond geometry (Å, °)

**Table:**

*D*—H···*A*	*D*—H	H···*A*	*D*···*A*	*D*—H···*A*
O2—H2···O3	0.82	1.87	2.686 (3)	172

## References

[bb1] Farrugia, L. J. (1997). *J. Appl. Cryst.* **30**, 565.

[bb2] Li, C.-J., Li, W., Tong, M.-L. & Ng, S. W. (2005). *Acta Cryst.* E**61**, m232–m234.

[bb3] Oxford Diffraction (2006). *CrysAlis CCD* and *CrysAlis RED* Oxford Diffraction, Abingdon, England.

[bb4] Reinoso, S., Vitoria, P., San Felices, L., Lezama, L. & Gutiérrez-Zorrilla, J. M. (2003). *Acta Cryst.* E**59**, m548–m550.

[bb5] Serna, Z., Barandika, M. G., Cortés, R., Urtiaga, M. K. & Arriortua, M. I. (1999). *Polyhedron*, **18**, 249–255.

[bb7] Sheldrick, G. M. (2008). *Acta Cryst.* A**64**, 112–122. 10.1107/S0108767307043930 18156677

[bb8] Tangoulis, V., Raptopoulou, C. P., Terzis, A., Paschalidou, S., Perlepes, S. P. & Bakalbassis, E. G. (1997). *Inorg. Chem. ***36**, 3996–4006.

[bb9] Tong, M.-L., Yang, G., Chen, X.-M. & Ng, S. W. (1998). *Acta Cryst.* C**54**, 217–219.

[bb10] Wang, S. L., Richardson, J. W., Briggs, S. J. & Jacobson, R. A. (1986). *Inorg. Chim. Acta*, **111**, 67–68.

[bb11] Yang, G., Tong, M.-L., Chen, X.-M. & Ng, S. W. (1998). *Acta Cryst.* C**54**, 732–734.

